# Synergistic and Additive Interactions of Zhongshengmycin to the Chemical Insecticide Pymetrozine for Controlling *Nilaparvata lugens* (Hemiptera: Delphacidae)

**DOI:** 10.3389/fphys.2022.875610

**Published:** 2022-05-30

**Authors:** Ruoying Zhao, Danting Li, Xinlong Wang, Zhong Li, Xiaoping Yu, Xuping Shentu

**Affiliations:** ^1^ Zhejiang Provincial Key Laboratory of Biometrology and Inspection and Quarantine, College of Life Science, China Jiliang University, Hangzhou, China; ^2^ Zhejiang Tonglu Huifeng Biosciences Co., Ltd., Hangzhou, China

**Keywords:** *Nilaparvata lugens*, chemical insecticide, antimicrobials, combination, synergistic effect

## Abstract

Management of the rice brown planthopper *Nilaparvata lugens* Stål is challenging because it can rapidly adapt to new pesticides within several generations. Combined use of chemical insecticides and antimicrobials was proposed as an alternative strategy to control *N. lugens*. Our previous experiments identified two effective agents (chemical insecticide: pymetrozine and antimicrobial: zhongshengmycin) that act on different targets in *N. lugens*. However, conditions and effectiveness of combinations of antimicrobials and insecticides against *N. lugens* are still unknown. Here, we evaluated separate and combined effects of pymetrozine and zhongshengmycin on third instar nymphs of *N. lugens* under laboratory and greenhouse conditions. Results showed that zhongshengmycin exerts significant inhibitory effects on the three endosymbionts *Pichia guilliermondii*, *Cryptococcus peneaus*, and *Pichia anomala* cultured *in vitro* of *N. lugens*. Combinations of pymetrozine and zhongshengmycin under laboratory conditions produced additive or synergistic effects on *N. lugens* and caused higher mortality in third instar nymphs than either of them used alone. Experiments under greenhouse conditions further demonstrated that effective component quality ratio of pymetrozine to zhongshengmycin of 1:10 and 1:40 with co-toxicity coefficients of 221.63 and 672.87, respectively, also produced significant synergistic effects against *N. lugens*. Our results indicated that chemical insecticides combined with antimicrobials may provide a potential novel strategy for controlling *N. lugens* by inhibiting its endosymbionts.

## 1 Introduction

The brown planthopper *Nilaparvata lugens* Stål (Hemiptera: Delphacidae) is a migratory and monophagous destructive pest of rice herbivore (Cheng et al., 2013). *N. lugens* obtains necessary nutrients from phloem sap of rice plant leaf sheath, causes indirect damage to rice plants through transmission of plant viruses in South and East Asia rice cultivation regions, and results in major yield reduction and economic losses (Garrood et al., 2016; Yang et al., 2017). Although chemical pesticides are still the main control strategy for *N. lugens*, this approach inevitably leads to the development of insecticide resistance, insect resurgence, and toxicity to natural enemies (Hu et al., 2014). Moreover, the time for *N. lugens* to develop resistance to common agents has remarkably reduced due to extensive and irregular use of chemical insecticides.

Therefore, developing increasingly effective control methods for the integrated management of *N. lugens*, which is closely related to people’s livelihood and environmental protection, is important. As it is known to us, endosymbionts include fungi and bacteria are ubiquitous in insects (Li et al., 2016). Endosymbionts have evolved from an organism into a kind of organelle-like structure in the long process of coevolution. A large number of studies have shown that *N. lugens* and endosymbionts form a stable mutualistic symbiosis, mutual dependence, and influence relationship due to their irreplaceable functional requirements (Sapp, 2002). Previous studies on endosymbionts of *N. lugens* mainly focused on diversity and dynamics of microbial communities at different developmental stages or feeding on different resistant rice varieties and their role in mediating host resistance (Cai et al., 2020; Jin et al., 2021; Zhang et al., 2021). Synergy effect of antimicrobials and insecticides on *N. lugens* population should be further explored given the close relationship between *N. lugens* and endosymbionts*.* The entire genome sequencing of *N. lugens* and its endosymbionts revealed that complementarity of three genomes with regard to nutritional pathways, including essential amino acid and steroid biosynthesis by the fungal symbiont and vitamin B supplementation by the bacterial symbiont, enables *N. lugens* to thrive on a low-nutrient diet provided solely by rice (Xue et al., 2014; Shi et al., 2021). The 3D reconstruction of *N. lugens* indicated that three structurally different endosymbionts, namely, yeast-like symbionts (YLSs), thread-, and rod-like bacterial symbionts, residing in fat body mycetocytes or midgut account for more than 22% of the abdominal volume of *N. lugens* (Song et al., 2021; Wang et al., 2021). Hence, endosymbionts are vital to the growth of *N. lugens* and may be potential targets of pest control.

Pymetrozine, a representative of pyridine azomethine compounds, is a chemical insecticide that exerts a significant control effect on specific species of stinging and sucking pest (Maienfisch et al., 2012; Li et al., 2021). Pymetrozine can be used to control aphids, leafhoppers, *Bemisia tabacis*, and rice planthoppers due to its high selectivity while showing safety to biological natural enemies of rice planthoppers, such as spiders and *Anagrus nilaparvataes*, during the control process (Preetha et al., 2010; Lin et al., 2021). The resistance of *N. lugens* to pymetrozine, sulfoxaflflor, nitenpyram, ect chemical insecticides has increased evidently in recent years (Liao et al., 2019; Zhang et al., 2021). Some field populations of *N. lugens* in China still remained susceptible to pymetrozine in 2010, but the resistance increased to medium or high levels in 2011 and 2012 (Zhang et al., 2014). The trend of increasing pymetrozine resistance was associated with irrational use of insecticide doses against rice planthoppers by managers and over-reliance on the use of chemical insecticides, such as pymetrozine, in Southeast Asia given that rice planthoppers in China typically migrate from Southeast Asia (Wang et al., 2013). Exploring improved methods for controlling and managing rice planthoppers is urgently necessary to avoid resistance development of *N. lugens* to additional types of chemical insecticides.

Zhongshengmycin is a broad-spectrum agro-antibiotic that has been investigated and developed in 1996 and demonstrated satisfactory effects on preventing and controlling crop pathogenic microorganisms, such as *Xanthomonas campestris*, *Erwinia carotovora, Piricularia oryzae, Pseudomonas solanacearum*, and *Physalospora piricola* (Xie et al., 1990; Zhao et al., 1993; Jiang et al., 1997). Zhongshengmycin works by interrupting the synthesis of protein peptide bonds of pathogenic microorganisms to inhibit the growth of bacteria, fungal mycelium, and spore germination (Yao et al., 2019). The successful exploitation of zhongshengmycin has replaced the use of some conventional chemical pesticides to a certain extent and presented high potential for common usage and application (Lin et al., 1991; Zhang et al., 1998). Therefore, the synergistic use of agro-antibiotics and chemical insecticides is an important strategy for pest control by inhibiting different targets, microbial endosymbionts and insects, respectively (Singh et al., 2016). Previous studies showed that the combination of pymetrozine and buprofezin 25% suspending agent is effective against *Sogatella furcifera* and *N. lugens* at the nymphal stage (Xing et al., 2011). Moreover, toxicity tests indicated that mixtures of imidacloprid and ethofenprox as well as thiamethoxam and ethofenprox exert excellent synergistic effects on *N. lugens* (Yu et al., 2015). The interaction between amitraz and malathion on *Aphis gossypii* presents a synergistic effect at all concentrations (Shojaei et al., 2018). Furthermore, a significant synergistic effect was observed when the mixture of destruxins and botanical insecticide rotenone was used at a concentration ratio of 9:1 in *A. gossypii* control (Yi et al., 2012). Accordingly, antimicrobials mixed with chemical agents may be a valuable strategy for pest control and provide an effective way of reducing the amount of chemical pesticides applied to crops while retarding resistance development of pests. The utilization of appropriately selected insecticides in association with antimicrobials can inhibit endosymbiont growth and result in synergistic and additive effects on *N. lugens* control.

Thus, we hypothesized in the present study that the combination of pymetrozine and zhongshengmycin at different concentration ratios may be effective against *N. lugens*. Our objective was to test whether combined effects of pymetrozine and zhongshengmycin were synergistic, additive, or antagonistic on *N. lugens* and obtain the optimal combination ratio of the mixture for the control of *N. lugens.* Furthermore, the optimal combination was used to test the mortality of *N. lugens* third instar nymphs under greenhouse conditions*.*

## 2 Materials and Methods

### 2.1 Insect and Antimicrobials

The rice variety used in this study was the susceptible strain TN1. Rice seeds were sown in the artificial climate room, and *N. lugens* was raised from rice seedlings in the tillering period. The *N. lugens* population used in this study was initially collected from rice fields in Hangzhou, China (continuously cultivated for more than ten generations under greenhouse conditions). Insects were reared on TN1 rice seedlings in an artificial climate chamber under conditions of 26 ± 1°C, relative humidity of 70%–80%, continuous 16 h light/8 h dark photoperiod, and nonexposure to any insecticide. Pymetrozine [96.6% active ingredient (a.i.) w/w] was purchased from Jiangsu Weunite Fine Co., Ltd. Zhongshengmycin (12% a.i. w/w) was provided by Fujian Kaili Biotechnology Co., Ltd. Tebuconazole (97% a.i. w/w) was obtained from Udragon Chemical Co., Ltd. N, N-Dimethylformamide (99.9%) was supplied by Shanghai Aladdin Bio-Chem Technology Co., Ltd.

### 2.2 Inhibitory Effect of Antimicrobials on Yeast-Like Symbionts

*Pichia guilliermondii*, *Cryptococcus peneaus,* and *P. anomala* strains were originally isolated from the fat body of *N. lugens* and preserved in Zhejiang Provincial Key Laboratory of Biometrology and Inspection and Quarantine. These isolates were activated in a plate of potato dextrose agar (PDA; 200 g of potato, 20 g of dextrose, and 20 g of agar in 1 L of distilled water) at 28°C ± 1°C. YLS suspensions were prepared by scraping YLS from the surface of the culture medium into a sterile PD solution (PD, 200 g of potato and 20 g of dextrose in 1 L of distilled water). Inhibitory effects of antimicrobials were determined by spreading YLS suspensions onto PDA plates containing various antimicrobial concentrations, followed by incubation at 28°C for an indicated duration.

### 2.3 Toxicity of Antimicrobials and Insecticides to *N. lugens*

Indoor toxicity testing of third instar nymphs of *N. lugens* was performed using rice seedling dip method (Ban et al., 2012; Liao et al., 2017). LC_50_ values of pymetrozine and zhongshengmycin against third instar nymphs were determined with a technical regulation method described previously [NY/T 1708-2009 technological rules for monitoring insecticide resistance in the rice brown planthopper *Nilaparvata lugens* (Stål)]. First, rice plants at the tillering stage were cut to a height of 10 cm. Second, three stems of rice plants were dipped into a plastic cup (10 cm in diameter and 18 cm in height) with a series of diluted solutions of pymetrozine and zhongshengmycin for 30 s and then placed rice stems in another test cup after air dried in the room temperature. Each insecticide or antimicrobial was diluted in six concentrations. Controls were treated with 0.1% Tween 80 water solution instead of the insecticide solution. Third, 20 third instar nymphs were collected into the test cup with a homemade aspirator device. All treatments were maintained at 26 ± 1°C and relative humidity of 70%–80% with a photoperiod of 16:8 (L:D) h. Each treatment was conducted in three independent biological replicates. Mortality of insects was monitored daily for 5 days after exposure. Nymphs were considered dead when they failed to move after prodding gently with a fine brush.

### 2.4 Screening of the Compound Proportion of Pymetrozine and Zhongshengmycin

On the basis of the toxicity bioassay and results of Wu and Si (2006), the toxic effect of different proportions of pymetrozine and zhongshengmycin on *N. lugens* was measured in this experiment. The synergistic prescription was selected according to laboratory experiments via interactive measurement. According to the results of medial lethal concentrations and toxicity measurement of pymetrozine and zhongshengmycin, toxic effect ratios of the two agents were set using 11 concentration gradients to determine the optimal compound proportion. 30 third instar nymphs were tested at each treatment concentration. Each treatment was conducted in three independent biological replicates. Expected mortality and toxic effect ratio are expressed as follows:

Expected mortality = M_a_ × the volume proportion of agent A in the mixture + M_b_ × the volume proportion of agent B in the mixture.

Note: M_a_: the observed mortality caused by agent A alone.

M_b_: the observed mortality caused by agent B alone.

The concertration of agent A and agent B were both LC_50_ (median lethal concentration) on test insects

$$\text{Toxic effect ratio}=\frac{\text{Observed Mortality}}{\text{Expected Mortality}}$$

### 2.5 Determining the Co-Toxicity Coefficient of Pymetrozine and Zhongshengmycin Compound

On the basis of the toxic effect ratio results, combinations with a toxic effect ratio greater than 1.25 were selected to show a synergistic effect for the determination of the co-toxicity coefficient of the optimal combination. Antagonism exists when the toxic effect ratio was less than 0.75. An additive effect was observed when the toxic effect ratio was approximately equal to 1.00. Experiments were conducted according to the procedure in Section 2.3. Each concentration treatment was repeated three times. Insect mortalities were recorded after 120 h. The co-toxicity coefficient, toxicity regression curve and its standard error, LC_50_ value, and confidence interval of 95% were determined on the basis of standard probit analysis via DPS 7.05. Toxicity index, actual toxicity index, theoretical toxicity index, and cotoxicity coefficient can be expressed as follows:

$$\text{Toxicity index }\left(\text{TI}\right)\text{ of single agent}=\frac{{\text{ LC}}_{50}\text{ of standard insecticide }}{{\text{ LC}}_{50}\text{ of single agent for testing}}\times 100$$

$$\mathrm{Actual}\;\mathrm{toxicity}\;\mathrm{index}\left(\mathrm{ATI}\right)\;\mathrm{of}\;\mathrm{mixtur}e_{\left(A+B\right)}=\frac{{\text{LC}}_{50}\text{ of standard insecticide}}{{\text{ LC}}_{50}\text{ of mixture}\left(\text{A}+\text{B}\right)}\times 100$$

Theoretical toxicity index (TTI) of mixture _(A+B)_ = TI_A_×mass percentage of agent A in the mixture + TI_B_×mass percentage of agent B in the mixture.

$$\text{Cotoxicity coefficient }\left(\text{CTC}\right)=\frac{\text{ATI }\left(\text{A}+\text{B}\right)}{\text{TTI }\left(\text{A}+\text{B}\right)}\times 100.$$

Note: standard insecticide: the single agent with a larger LC_50_ was used as the standard insecticide, and its toxicity index was regarded as 100.

$$TI_{A}:\;\text{toxicity index of agent A}$$

$$TI_{B}:\;\;\text{toxicity index of agent B}$$

$$\mathrm{AT}I_{\left(A+B\right)}:\;\text{actual toxicity index}\;\text{of }\;\mathrm{mixtur}e_{\left(A+B\right)}$$

$${\text{TTI }}_{\left(\text{A}+\text{B}\right)}{\text{: theoretical toxicity index of mixture}}_{\left(\text{A}+\text{B}\right)}$$

### 2.6 Data Analysis

Bioassay data were analyzed with DPS 7.05. Mortality data were corrected using the control mortality of Abbott’s formula (Richard and Arthur, 1985). LC_50_ values, 95% confidence intervals (CI), slopes of regression lines, standard errors, and other relevant data were estimated via probit analysis (Finney, 1971). Treatment effects on mortality levels were assessed using one-way ANOVA. Differences between treatments were deemed significant when *p* < 0.05. A synergistic effect exists between the two pesticides when the toxic effect ratio of measured insecticides was greater than 1.25. Antagonism exists when the toxic effect ratio was less than 0.75. An additive effect exists when the toxic effect ratio was approximately equal to 1.00. The proportion of compounding agents with a toxic effect ratio greater than 1.25 was selected for indoor toxicity determination, and the toxicity regression curve and LC_50_ value of the compound were further obtained. The toxicity index of the single agent and theoretical toxicity index, actual toxicity index, and co-toxicity coefficient of the compound were calculated using the method of Sun Yun-pei (Sun and Johnson, 1960). Compounding agents were mutually synergistic when the cotoxicity coefficient was greater than 120. The interaction was additive when the cotoxicity coefficient was between 80 and 120. An antagonistic effect was observed when the cotoxicity coefficient was less than 80.

## 3 Results

### 3.1 Inhibitory Effect of Different Fungicides on the Yeast-Like Symbiont

**FIGURE 1:**
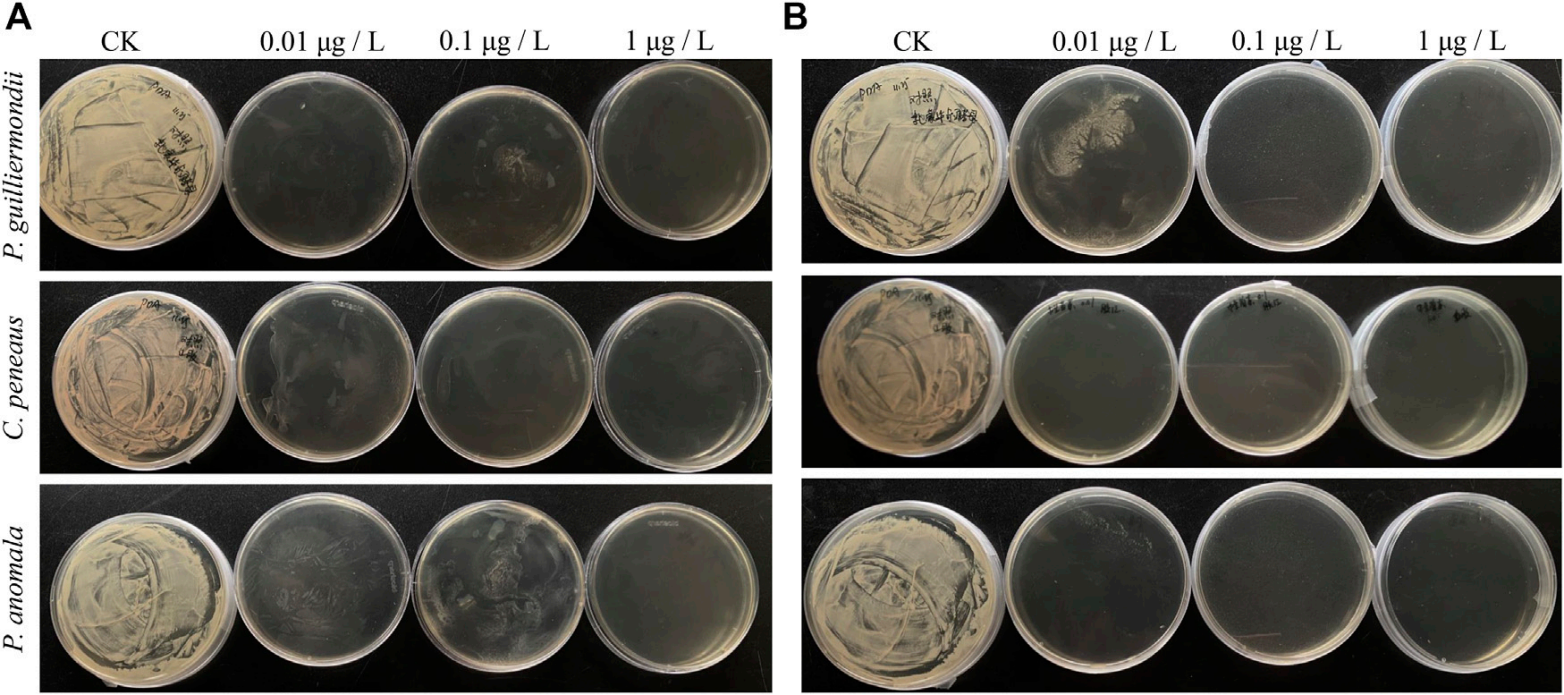
The growth of YLSs isolated from *Nilaparvata lugens* at different concentrations of antimicrobials. **(A)** Tebuconazole; **(B)** Zhongshengmycin.

Table 1 and Figure 1 showed the growth of *P. guilliermondii, C. peneaus*, and *P. anomala* under different concentrations of tebuconazole and zhongshengmycin on PDA medium. First, *P. guilliermondii*, *C. peneaus*, and *P. anomala* all showed significant growth on the PDA control plate but negative on two kinds of PDA plates with concentrations of 1 μg/L tebuconazole or zhongshengmycin. Second, *P. guilliermondii* and *C. peneaus* presented a small amount of growth on the PDA plate with 0.01 and 0.1 μg/L of tebuconazole. Third, *P. guilliermondii, C. peneaus*, and *P. anomala* demonstrated major, negative and minimal growth on the PDA plate with 0.01 μg/L of zhongshengmycin, respectively. No or nearly no growth was observed on PDA plates with 0.1 μg/L of zhongshengmycin.

**TABLE 1:** Inhibitory effect of different antimicrobials on YLS isolated from *Nilaparvata lugens*.

Antimicrobials	*Pichia guilliermondii*				*Cryptococcus peneaus*				*Pichia anomala*			
Concentration (μg/L)	0	0.01	0.1	1.0	0	0.01	0.1	1.0	0	0.01	0.1	1.0
Tebuconazole	+++	+	+	−	+++	+	+	−	+++	+	++	−
Zhongshengmycin	+++	++	−	−	+++	−	−	−	+++	+	−	−

+++: Vast growth, ++: Major growth, +: Minimal growth, and −: Negative growth.

Different concentrations of Zhongshengmycin generally demonstrated better inhibition effect on *P. guilliermondii, C. peneaus*, and *P. anomala* than those of tebuconazole. Therefore, Zhongshengmycin may present optimal efficiency in controlling *N. lugens* by inhibiting its endosymbionts.

### 3.2 Median Lethal Concentration of Pymetrozine and Zhongshengmycin

As shown in Table 2, pymetrozine and zhongshengmycin present high biological activity to third instar nymphs and the relationship between nymph mortality and insecticide concentration is clearly described through the probit model. Estimated LC_50_ values of pymetrozine and zhongshengmycin for third instar nymphs of *N. lugens* were 3.80 (95% confidence interval: 1.84–7.85 mg/L) and 152.61 (95% confidence interval: 45.34–513.70 mg/L) mg/L while LC_95_ values were estimated at 113.04 (95% confidence interval: 38.73–329.94 mg/L) and 895.00 (95% confidence interval: 262.10–3056.18 mg/L) mg/L, respectively, at 5 days after application.

**TABLE 2:** Median lethal concentrations of pymetrozine and zhongshengmycin on third instar nymphs of *Nilaparvata lugens*.

Antimicrobials	Probit equation	Standard error	χ^2^ (df)	LC_50_ value mg ai/L (95% CI)	LC_95_ value mg ai/L (95% CI)	*p* value
96.6% Pymetrozine	Y = 4.3532 + 1.1161x	0.17	0.24 (8)	3.80 (1.84 − 7.85)	113.04 (38.73 − 329.94)	0.0076
12% Zhongshengmycin	Y = 0.3247 + 2.1411x	0.55	1.28 (8)	152.61 (45.34 − 513.70)	895.00 (262.10 − 3056.18)	0.0295

### 3.3 Effect of Interaction Between Pymetrozine and Zhongshengmycin

The proportion screening of pymetrozine and zhongshengmycin in the mixture was carried out through toxic effect ratio (Table 3). The combination of pymetrozine and zhongshengmycin demonstrated a synergistic effect on third instar nymphs, with toxic effect ratios of 1.28 and 1.29, respectively, in groups 3 and 6. Groups 2, 4, 5, 7, 8, 9, and 10 showed additive effects with the toxic effect ratio between 1 and 1.25. Hence, combinations of pymetrozine and zhongshengmycin targeting third-instar nymphs of *N. lugens* showed synergistic and additive effects without antagonism. The mortality of all combination treatments was higher than that of individual treatments with zhongshengmycin alone; when compared with pymetrozine alone, the mortality of the combination treatments was partially higher than that of the individual treatments. The treatment results showed that at some ratio of combination with zhongshengmycin and pymetrozine had better control effect than that of zhongshengmycin or pymetrozine alone. Overall, these findings indicated that combinations of pymetrozine and zhongshengmycin increase the mortality of *N. lugens*. Furthermore, groups 3 and 6 with the synergistic effect are selected for indoor toxicity determination through the comparison of toxic effect ratios.

**TABLE 3:** Interactions between pymetrozine and zhongshengmycin against third instar nymphs of *Nilaparvata lugens* 5 days after treatment.

Treatment	Py:Zs	Number	Death number^a^	Observed mortality (%)±SE	Expected mortality^b^ (%)	Toxic effect ratio^c^ (120 h)
1	100:0	30	20	66.67 ± 0.27^a^ ^,^ ^b^ ^,^ ^c^ ^,d^		
2	90:10	30	23	76.67 ± 0.09^a^ ^,^ ^b^	64.67	1.19
3	80:20	30	24	80.00 ± 0.13^a^	62.67	1.28
4	70:30	30	21	70.00 ± 0.22^a^ ^,^ ^b^ ^,^ ^c^	60.67	1.15
5	60:40	30	18	60.00 ± 0.39^c^ ^,d,e^	58.67	1.02
6	50:50	30	22	73.33 ± 0.11^a^ ^,^ ^b^ ^,^ ^c^	56.67	1.29
7	40:60	30	18	60.00 ± 0.42^b^ ^,^ ^c^ ^,d,e^	54.67	1.10
8	30:70	30	17	56.67 ± 0.38^c^ ^,d,e^	52.67	1.08
9	20:80	30	18	60.00 ± 0.17^b^ ^,^ ^c^ ^,d,e^	50.67	1.18
10	10:90	30	15	50.00 ± 0.42^d,e^	48.67	1.03
11	0:100	30	14	46.67 ± 0.27^e^		
12	CK	30	0	0^f^		

Note : Py, Pymetrozine and Zs, Zhongshengmycin (The ratio of Py:Zs represent volume ratio).

a Mean values of three replicates of the experiment. Different lowercase letters indicate significant differences among different treatments in the same day after application (*p* < 0.05, Tukey’s LSD test).

b Expected mortality = M_a_ × the volume proportion of agent A in the mixture + M_b_ × the volume proportion of agent B in the mixture.

M_a_: the observed mortality caused by agent A alone, M_b_: the observed mortality caused by agent B alone, The concertration of agent A and agent B were both LC_50_ (median lethal concentration) on test insects.

c Toxic effect ratio = observed mortality/Expected Mortality.

Significant differences were indicated by different letters at *p* < 0.05.

### 3.4 Combined Effects of Pymetrozine and Zhongshengmycin Against *N. lugens* Under Greenhouse Conditions

Tables 3, 4 indicate that the effective component quality ratio of group 3 (pymetrozine:zhongshengmycin = 80:20) is the combination of pymetrozine: zhongshengmycin = 1:10 for laboratory toxicity determination. LC_50_ of the combination to third instar nymphs was 15.1012 mg/L (95% confidence interval: 8.6673–26.3113 mg/L) and co-toxicity coefficient was 221.63 at the effective component quality ratio of pymetrozine and zhongshengmycin was 1:10. LC_50_ of the combination to third instar nymphs was 11.6010 mg/L (95% confidence interval: 6.6270–20.3082 mg/L) and co-toxicity coefficient was 672.87 at the effective component quality ratio of pymetrozine and zhongshengmycin was 1:40. Co-toxicity coefficients of the two combinations with different effective component quality ratios were all greater than 120, thereby indicating the significant synergistic effect. On the basis of these experimental results, treatments with combinations of pymetrozine and zhongshengmycin resulted in higher mortality and the formation of a synergistic effect compared with treatments with insecticide or fungicide alone.

**TABLE 4:** Determination of the cotoxicity coefficient of pymetrozine and zhongshengmycin on third instar nymphs of *N. lugens* and statistical results of the adjustment to the log-probit model under greenhouse condition.

Treatment (Py:Zs)	Probit equation	LC_50_ value (mg ai/L)	LC_50_ value mg ai/L (95% CI)	*p* value	*r*	CTC
1:10	Y = 5.6708 + 0.3684x	15.1012	8.66–26.31	0.0039	0.9780	221.63
1:40	Y = 6.6181 + 0.8360x	11.6010	6.62–20.30	0.0028	0.9824	672.87

Py, pymetrozine; Zs, Zhongshengmycin; CTC, Cotoxicity coefficient.

## 4 Discussion

Pymetrozine is one of the recommended alternative insecticides for controlling *N. lugens*. However, *N. lugens* has become highly resistant to pymetrozine (Liu et al., 2013; Zhu et al., 2020). Combined effects of pymetrozine and an antimicrobial (zhongshengmycin) on nymphs of *N. lugens* were investigated in the present study to delay pymetrozine resistance development and prolong the effective application of this insecticide. Our findings indicated that all combined groups exert synergistic or additive effects on the control of *N. lugens*. The mortality of third-instar nymphs increased in the combinations treatment of pymetrozine and zhongshengmycin, while compared with that of the treatments used pymetrozine and zhongshengmycin alone. Additionally, the effective component quality ratio of pymetrozine and zhongshengmycin of 1:10 and 1:40 proved to have significant synergistic effects against third instar nymphs under greenhouse conditions by calculating the co-toxicity coefficient. Compared with chemical insecticides, microbial metabolites present characteristics of lower toxicity and lower environmental pollution coefficient (Chen et al., 2009; Cao et al., 2015). Therefore, the synergistic use of antimicrobials and chemical insecticides is a safe, efficient, and potential approach for pest management for presenting advantages of obvious biocontrol effect and reduced use of chemical insecticides.

The use of alternative substances to chemical insecticides has attracted considerable research interest due to its lowered risk to the environment and human health and increased food safety. Combinations of antimicrobials and insecticides can exhibit synergistic, antagonistic, or additive effects on pests. For example, combined application of *Serratia marcescens* S-JS1 with spirotetramat or thiamethoxam resulted in increased effcacy against *N. lugens* under both laboratory and greenhouse conditions (Niu et al., 2018). In addition, positive synergistic interactions in combined treatments of pymetrozine and thiamethoxam against *N. lugens* revealed that the controlling effect of combined treatments on rice planthoppers is better than that of the commercial insecticide 2.2% abamectin–imidacloprid emulsifiable concentrate at comparable doses (Sadigh and Erhart, 2012). Willmott et al. (2013) reported that spinosad and pymetrozine mixtures are clearly compatible and combination index calculations showed that mixtures are synergistic against western flower thrips. Cao et al. (2015) revealed that 70% propineb and 50% tebuconazole+25% trifloxystrobin exert evident inhibitory effects on *P. anomala* isolated from *Laodelphax striatellus in vitro* and *L. striatellus* fed with treated wheat seedlings show significantly higher mortality than the control. Controlling *N. lugens* with fungicides has been extensively investigated. For instance, injection of propiconazole into *N. lugens* reduced not only fecundity of insects but also significantly reduced the total number of YLS and *Hypomyces chrysospermus* in hemolymph of insects. This finding led to significantly higher mortality of *N. lugens* than that of the control group. Survival and fecundity of *N. lugens* also decreased significantly after feeding on susceptible species TN1 sprayed with propiconazole (Shentu et al., 2019). Moreover, high mortlity of *N. lugens* due to the administration of 27% toyocamycin + tetramycin P + tetrin B + tetramycin A, 0.01% trichodermin, and 75% trifloxystrobin + tebuconazole WG inhibited the YLS (Shentu et al., 2016). The application of another fungicide jinggangmycin for controlling rice sheath blight can successfully inhibit the reproduction of *L. striatellus* and *S. furcifera* but also stimulate the reproduction of *N. lugens* (Zhang et al., 2015; Ding et al., 2017). However, we provided a new combined formulation of pymetrozine and zhongshengmycin for *N. lugens* control. Our results have showed that two combinations of pymetrozine and zhongshengmycin at the effective component quality ratio of 1:10 and 1:40 have significant synergistic effects on the control of *N. lugens*. The mixing ratio and resulting economic benefits should be considered in practical applications given that the two combined groups demonstrated significant synergistic effects against *N. lugens*. Furthermore, reasons behind the ability of combined pymetrozine with zhongshengmycin to produce synergistic or additive effects on *N. lugens* are presented in the following section.

Applying combinations of antimicrobials with insecticides may improve not only the efficacy of agents but also provide a potential strategy for reducing insecticide use in pest control (Lacey et al., 2015). Zhongshengmycin exerted a significant inhibitory effect on *P. guilliermondii*, *C. peneaus*, and *P. anomala*, which were isolated from *N. lugens* within 1–3 days of *in vitro* culture in the present study. We hypothesized that zhongshengmycin can help control *N. lugens* by inhibiting endosymbionts, which play a vital role in the growth, development, and reproduction of their host insects. However, difference may exist in the inhibitory effect on YLSs between *in vivo* and *in vitro* cultures. Moreover, *in vitro* culturing of many kinds of symbionts in *N. lugens*, including endosymbiotic bacteria and YLSs, is impossible. Determining whether zhongshengmycin exerts inhibitory effects on uncultured symbionts is challenging from an evolutionary perspective because important symbionts are difficult to culture *in vitro*. Therefore, the specific kind of YLS in *N. lugens* inhibited by zhongshengmycin requires further investigation.

In conclusion, our study emphasized the importance of combining chemical insecticides with antimicrobials for the control of *N. lugens* by inhibiting endosymbionts under both laboratory and greenhouse conditions. We first screened out zhongshengmycin, an antimicrobial with strong inhibitory effect on YLS, in *N. lugens*. Then, we applied the combination of zhongshengmycin and pymetrozine to prevent *N. lugens* and verify that the increased mortality of *N. lugens* is caused by the inhibitory effect of the combination on YLSs in *N. lugens*. The results of this study addressed the efficacy gap of single agent treatment in *N. lugens* control and improved the efficacy in controlling *N. lugens* given that all test combinations produced additive or synergistic effects. We also revealed that synergistic and additive effects produced by the combinations of zhongshengmycin and pymetrozine may vary depending on their concentrations and types. This finding may provide an effective option for reducing concentrations and doses of chemical insecticides in the future. However, the exact function of the compound in symbionts of *N. lugens* remains unclear. We speculated that the function may be caused by the change in endosymbionts. Hence, further investigations on the number and function of these endosymbionts under different chemical insecticides or antimicrobials are necessary to address such problems. Understanding the effect of these chemical insecticides or antimicrobials on endosymbionts of *N. lugens* and using the combination of chemical insecticides and antimicrobials can be an important treatment for integrated pest management of *N. lugens* in the future.

## Data Availability

The original contributions presented in the study are included in the article/Supplementary Material, further inquiries can be directed to the corresponding authors.
